# Rapid Recovery of Acute Respiratory Distress Syndrome in Scrub Typhus, With Pulse Methylprednisolone and Therapeutic Plasma Exchange

**DOI:** 10.7759/cureus.30329

**Published:** 2022-10-15

**Authors:** Thilina Rathnasekara, Lanka Wijekoon, Hemal Senanayake, Sisira Siribaddana

**Affiliations:** 1 Internal Medicine, Postgraduate Institute of Medicine, University of Colombo, Colombo, LKA; 2 Medicine, Faculty of Medicine, University of Rajarata, Anuradhapura, LKA

**Keywords:** orientia tsutsugamushi, therapeutic plasma exchange, methylprednisolone pulse, acute respiratory distress syndrome, scrub typhus

## Abstract

Acute respiratory distress syndrome (ARDS) is an atypical presentation of scrub typhus infection with associated high morbidity and mortality compared to uncomplicated scrub typhus infection. Here we report an otherwise healthy 41-year-old female patient admitted with moderate ARDS secondary to scrub typhus infection who gained comparatively rapid recovery within 72 hours following intravenous methylprednisolone pulse (MPP) therapy with therapeutic plasma exchange (TPE), suggesting a beneficial role of MPP and TPE in the treatment of ARDS secondary to scrub typhus infection. To our knowledge, this is the first case report regarding the use of MPP therapy and TPE in a patient with ARDS due to scrub typhus infection.

## Introduction

Scrub typhus is a rickettsia-like zoonotic disease that presents as an acute febrile illness after the bite of an infected mite [1]. Other common presentations include headache, myalgia, generalized lymphadenopathy, cough, gastrointestinal symptoms, and transient hearing loss. Scrub typhus can be presented with atypical manifestations, including unilateral lower motor facial nerve palsy, acute respiratory distress syndrome (ARDS), meningoencephalitis, gastrointestinal bleeding, acute renal failure, and coagulopathy [2,3]. We report the case of a 41-year-old female who developed ARDS following a scrub typhus infection. She recovered rapidly following treatment with therapeutic plasma exchange (TPE) and methylprednisolone pulse (MPP).

## Case presentation

A 41-year-old previously healthy female farmer from a rural area in the Anuradhapura district, Sri Lanka, was admitted to a regional hospital with high fever, chills, rigors, arthralgia, and myalgia for three days. In addition, she had gradually developed a non-productive cough and breathing difficulty and was transferred to the teaching hospital in Anuradhapura for further management. On admission, she was conscious and rational but dyspnoeic in room air, and there was no history of hemoptysis. The SpO_2_ was 62% in room air with a respiratory rate of 40 breaths per minute. She had respiratory crackles in all three zones of both lungs.

**Figure 1:**
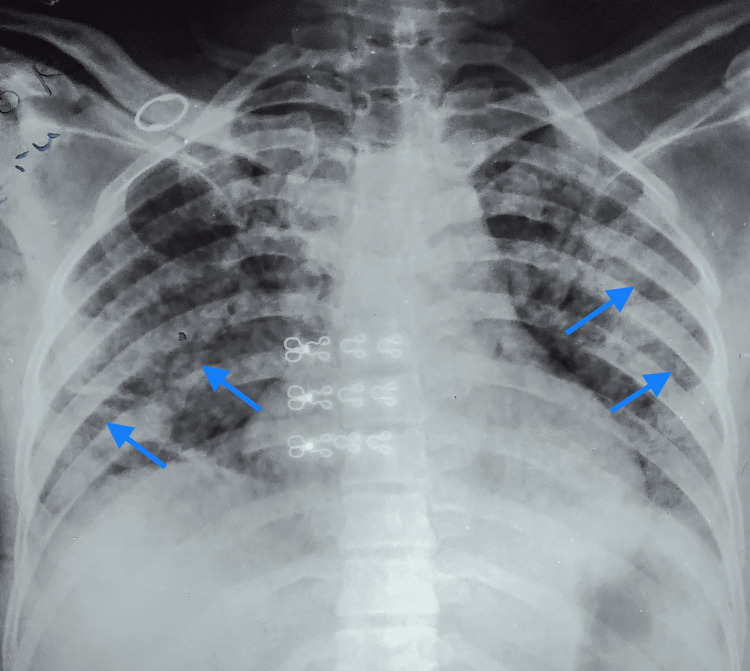
Chest x-ray on admission, showing bilateral non-homogenous opacities (blue arrows) without cardiomegaly consistent with ARDS. ARDS: acute respiratory distress syndrome.

Her arterial blood gas analysis in room air revealed a PaO_2_ of 34 mmHg and a PaO_2_/FiO_2_ ratio of 162 mmHg, suggesting moderate ARDS. Her chest x-ray showed bilateral non-homogeneous opacity (Figure 1). Non-invasive ventilation with continuous positive airway pressure (CPAP) was initiated. 

She had neutrophil leukocytosis (19.13 × 10^3^/uL with 82.3% neutrophils) and low platelets (62000/uL). Her clotting profile and serum creatinine were normal (56 umol/L), and she had marginal transaminitis (aspartate transaminase (AST) 73 U/L and alanine transaminase (ALT) 54 U/L) with high C-reactive protein (63.5 mg/l). 

**Figure 2:**
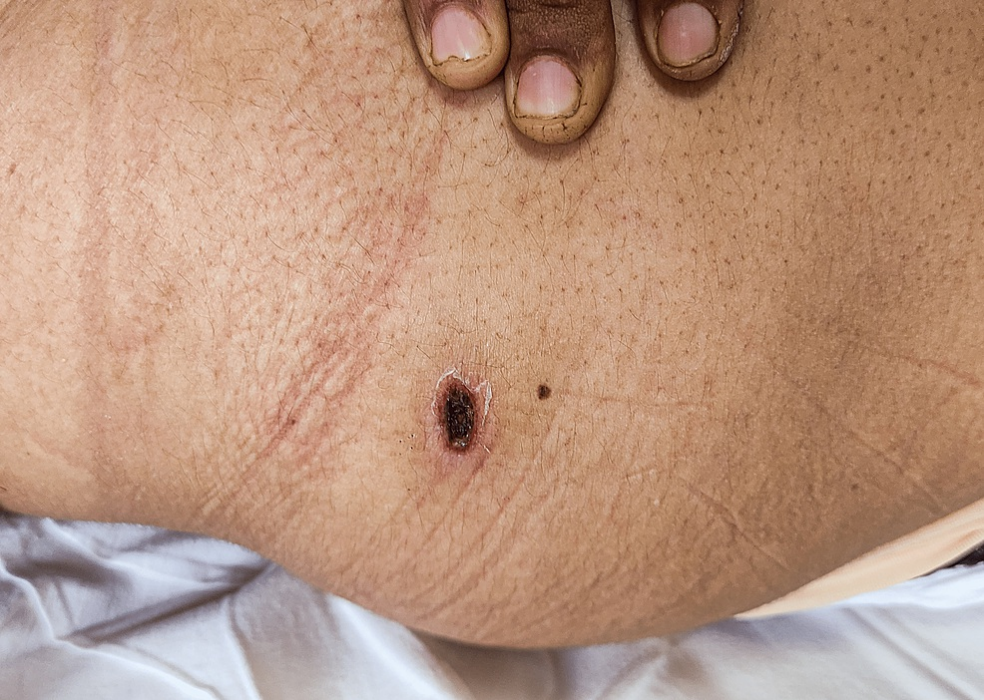
Eschar over the patient's right buttock region.

She was initially treated as a patient with severe leptospirosis with pulmonary involvement, for which intravenous third-generation cephalosporin (ceftriaxone) was commenced with MPP, and TPE was planned. The diagnosis of scrub typhus complicated by ARDS was made after a whole-body examination, which revealed an eschar in her right buttock (Figure 2).

Scrub typhus antibody IgM, an enzyme-linked immunosorbent assay (ELISA) based lateral flow immunochromatographic assay, was weakly positive on the fifth day of her illness. A repeat IgM done on the tenth day was positive, confirming the diagnosis of scrub typhus. Due to the unavailability, we could not perform a polymerase chain reaction (PCR) assay for scrub typhus. Her severe acute respiratory syndrome coronavirus 2 PCR test came as not detectable. Her blood and urine cultures yielded no growth, and her microscopic agglutination test for leptospirosis was negative.

ARDS in scrub typhus may be an immunopathological phenomenon. Hence, we proceeded with one cycle of TPE with the fresh frozen plasma of 2580 ml, normal saline, and calcium gluconate. Two doses of MPP 500 mg daily were administered with doxycycline 100 mg twice daily for fourteen days. She improved dramatically with this treatment. CPAP ventilation was given for only 24 hours, and oxygen was given through nasal prongs for the next 48 hours. After six days of in-hospital treatment, she was asymptomatic and discharged. At the follow-up visit after two weeks, she was completely asymptomatic. A timeline of the important events is presented in Table 1.

**Table 1:** Timeline of the patient’s clinical condition, investigations and interventions. ARDS: acute respiratory distress syndrome; CPAP: continuous positive airway pressure; CRP: C-reactive protein; MPP: methylprednisolone pulse; TPE: therapeutic plasma exchange.

Dates	Events
December 19, 2021	The patient was asymptomatic.
December 20, 2021	The patient developed a fever.
December 22, 2021	The patient developed breathing difficulties with a cough and was admitted to the regional hospital.
December 23, 2021	Due to dyspnea, the patient was transferred to the teaching hospital in Anuradhapura, and her chest x-ray revealed evidence of ARDS. We started her on CPAP ventilation due to moderate ARDS, and blood investigations revealed neutrophil leukocytosis, thrombocytopenia, marginal transaminitis, and elevated CRP. We gave the first dose of MPP and TPE on the same day.
December 24, 2021	We gave the second dose of MPP. IgM for scrub typhus was weakly positive. We de-escalated the patient from CPAP to nasal prong oxygen.
December 26, 2021	We omitted the supplementary oxygen.
December 29, 2021	Repeat IgM for scrub typhus was positive, and we discharged the patient.
January 12, 2022	The patient was completely asymptomatic.

## Discussion

Scrub typhus is a zoonotic disease caused by the bacteria *Orientia tsutsugamushi*, a common rickettsia-like illness in Sri Lanka. Infected trombiculid mites of the genus *Leptotrombidium* act as definitive hosts or natural reservoirs. Humans can accidentally get infected when bitten by an infected larva of a trombiculid mite (chigger) [1].

*O. tsutsugamushi* is an obligatory intracellular gram-negative bacterium that can multiply within the endothelial cells of small vessels in humans. Systemic vasculitis and perivasculitis are the pathological hallmarks of scrub typhus, causing heightened vascular permeability and hypo-perfusion [3].

Disease manifestation varies from a range of non-specific simple febrile illnesses to multi-organ dysfunction. The presence of a characteristic skin lesion, eschar, varies from 25% to 89% among patients with confirmed scrub typhus [1]. Lung involvement can be diverse, from uncomplicated bronchitis to severe acute respiratory distress syndrome [2,3].

Abnormal investigations in scrub typhus are elevated transaminases in 87%, thrombocytopenia in 79%, and leukocytosis in 46% [4], all of which were present in our patient. The indirect immunofluorescence assay is the gold standard for the diagnosis of scrub typhus. ELISA-based estimation of IgM antibodies is now preferred over indirect immunofluorescence assay [5]. This is because the indirect immunofluorescence assay is expensive with limited availability and has significant inter-operator variability.

Acute onset hypoxemia with bilateral pulmonary infiltrates that resemble pulmonary edema in the absence of left heart failure is classified as ARDS [6]. A retrospective study reported that the ARDS prevalence among scrub typhus is 1.7%, with a mortality rate of 36% [7]. The pathogenesis of ARDS in scrub typhus is poorly understood, but a cytokine-mediated immune response is suspected [8]. The activation of pulmonary endothelial cells is by direct infection or indirectly via inflammatory cytokines. A further increase in recruiting immune cells by secreted chemokines will occur, resulting in the breakdown of the endothelial barrier, causing pleural effusion and pulmonary edema, similar to what happens in a cytokine storm [8].

Oral doxycycline is the first-line treatment for uncomplicated scrub typhus infections. Other options include oral azithromycin and, in drug-resistant strains, oral rifampicin [9]. However, additional therapy is needed when complicated with ARDS. In a case report, ARDS associated with scrub typhus was treated with extracorporeal membrane oxygenation and antibiotics with hospitalization for 23 days [3]. Our patient was weaned off the CPAP after 24 hours and was sent home after six days.

The reason for the rapid improvement may be due to the MPP and TPE she received while on oral doxycycline, which may have helped to neutralize the immunological injury to the lungs. Due to the anti-inflammatory effects of corticosteroids, they can be used to treat conditions with an overactive inflammatory response. Prolonged corticosteroid treatment in early ARDS has demonstrated survival benefits [10]. TPE can promptly suppress the patient's immune response with a good safety profile and is currently used in leptospirosis complicated by pulmonary hemorrhage [11]. There is a case report of pulmonary hemorrhage due to leptospirosis and scrub typhus co-infection treated with TPE [12]. However, to our knowledge, there are no reported cases of using MPP and TPE to treat ARDS due to typhus infection.

## Conclusions

Scrub typhus infection is a rickettsia-like infection with many typical and atypical presentations. ARDS is one of the atypical presentations with significant morbidity and mortality. This report showed that using MPP and TPE in a patient with ARDS due to an infection with scrub typhus demonstrated promising results. This can be used to reduce associated morbidity and mortality by shortening the duration of the illness, even though the proof of the efficacy of such treatment remains anecdotal.
